# Acceptance of a COVID-19 Vaccine in Southeast Asia: A Cross-Sectional Study in Indonesia

**DOI:** 10.3389/fpubh.2020.00381

**Published:** 2020-07-14

**Authors:** Harapan Harapan, Abram L. Wagner, Amanda Yufika, Wira Winardi, Samsul Anwar, Alex Kurniawan Gan, Abdul Malik Setiawan, Yogambigai Rajamoorthy, Hizir Sofyan, Mudatsir Mudatsir

**Affiliations:** ^1^Medical Research Unit, School of Medicine, Universitas Syiah Kuala, Banda Aceh, Indonesia; ^2^Tropical Disease Centre, School of Medicine, Universitas Syiah Kuala, Banda Aceh, Indonesia; ^3^Department of Microbiology, School of Medicine, Universitas Syiah Kuala, Banda Aceh, Indonesia; ^4^Department of Epidemiology, University of Michigan, Ann Arbor, MI, United States; ^5^Department of Family Medicine, School of Medicine, Universitas Syiah Kuala, Banda Aceh, Indonesia; ^6^Department of Pulmonology and Respiratory Medicine, School of Medicine, Universitas Syiah Kuala, Banda Aceh, Indonesia; ^7^Department of Statistics, Faculty of Mathematics and Natural Sciences, Universitas Syiah Kuala, Banda Aceh, Indonesia; ^8^Department of Microbiology, Faculty of Medicine and Health Sciences, Maulana Malik Ibrahim State Islamic University of Malang, Malang, Indonesia; ^9^Department of Economics, Faculty of Accountancy and Management, Universiti Tunku Abdul Rahman, Cheras, Malaysia

**Keywords:** COVID-19, SARS-CoV-2, vaccine, vaccination, acceptance

## Abstract

**Introduction:** Several vaccine candidates are being clinically tested in response to the 2019 coronavirus disease (COVID-19) pandemic. This study was conducted to assess the acceptance of a 50 or 95% effective COVID-19 vaccine, when it becomes available in southeast Asia, among the general population in Indonesia.

**Methods:** A cross-sectional online survey was conducted between March 25 and April 6, 2020. Participants were asked if they would accept a free vaccine which was 95 or 50% effective. Using a logistic regression model, we assessed the associations between sociodemographic characteristics, exposure to COVID-19 information, or perceived risk of infection with acceptance of a hypothetical COVID-19 vaccine.

**Results:** Among 1,359 respondents, 93.3% of respondents (1,268/1,359) would like to be vaccinated for a 95% effective vaccine, but this acceptance decreased to 67.0% (911/1,359) for a vaccine with 50% effectiveness. For a 95% effective vaccine, being a healthcare worker and having a higher perceived risk of COVID-19 infection were associated with higher acceptance, adjusted odds ratio (aOR): 2.01; 95%CI: 1.01, 4.00 and aOR: 2.21; 95%CI: 1.07, 4.59, respectively; compared to civil servants, being retired was associated with less acceptance (aOR: 0.15; 95%CI: 0.04, 0.63). For a 50% effective vaccine, being a healthcare worker was also associated with greater acceptance, aOR: 1.57; 95%CI: 1.12, 2.20.

**Conclusion:** Acceptance of a COVID-19 vaccine was highly influenced by the baseline effectiveness of the vaccine. Preparing the general population to accept a vaccine with relatively low effectiveness may be difficult.

## Introduction

The current 2019 coronavirus disease (COVID-19) pandemic, caused by severe acute respiratory syndrome coronavirus 2 (SARS-CoV-2), is a major threat worldwide and especially to countries in southeast Asia (1–3). A systematic review of 53,000 hospitalized patients indicated that 20.2% of COVID-19 cases developed severe disease with a mortality rate of ~3.1% (4). In the elderly and among those with comorbidities, such as cardiovascular disease, chronic kidney disease, and chronic obstructive pulmonary disease, mortality increases significantly (5–8). Although some drugs have been used to treat severe COVID-19 patients (9–12), no specific therapies have been approved by the US Food and Drug Administration. Development and deployment of a vaccine is therefore one of the most promising strategies in this crisis.

Vaccine development began in several research centers and pharmaceutical companies as soon as SARS-CoV-2 was identified as the causative agent and the first genome sequence was published. On March 16, 2020, the first COVID-19 vaccine candidate, an mRNA-based vaccine developed by Moderna Inc, entered a Phase 1 clinical trial (NCT04283461) in the US and later a non-replicating vector-based vaccine, developed by China's CanSino Biologics was also tested in China (ChiCTR2000030906) (13). Other vaccine candidates, including DNA-based vaccines, inactivated, live attenuated, sub-unit, and replicating viral vector-based vaccines are also being developed (13). It is unclear how effective these vaccines will be. If the COVID-19 vaccine resembles an influenza vaccine, effectiveness could be 50% or lower (14). People may have strong preferences for a vaccine to be highly effective (15), and a vaccine with a low effectiveness estimate could impact people's willingness to be vaccinated. It is also possible that individuals will perceive a pandemic vaccine to be less safe based on its newness or perceived lack of testing (15). Safety perceptions could also influence vaccine acceptance (16).

High vaccination coverage globally may be required to stop the COVID-19 pandemic. However, vaccine demand in low- and middle-income countries (LMICs) is less well-studied and there may be different considerations from the population compared to high income countries (17). LMICs may have less capacity to introduce new vaccines and may need to deal with citizenry who have hesitant beliefs (18). Indonesia is a middle-income country with relatively low vaccine coverage and high vaccine hesitancy (18–20). Some studies have been conducted to assess acceptance on new vaccines against emerging and re-emerging infectious diseases in southeast Asia, such as for dengue (21–25), Zika (26), and Ebola (27). No study has been conducted on COVID-19 vaccine acceptance in the region. This study sought to assess the acceptance of a hypothetical COVID-19 vaccine among the general population in Indonesia. The results of this study might be important for the government to formulate the best approach to implement mass vaccination programs for COVID-19 in Indonesia, as well as other countries in southeast Asia region, in the future.

## Methods

### Study Design and Setting

Currently no COVID-19 vaccine is available and therefore we framed the study questions around a hypothetical vaccine, in an approach that was similar to previous studies (21, 26, 28–30). Due to limitations in doing face-to-face research during the current active COVID-19 outbreak in Indonesia, we did an online cross-sectional study between March 25 and April 6, 2020. The target population was the adult population of Indonesia. The samples were recruited from seven provinces (Aceh, West Sumatra, Jambi, DKI Jakarta, Yogyakarta, and Bali) and all adults who were able to read and understand Bahasa Indonesia were considered eligible. Invitations to participate in the study, hosted by Google Forms, were distributed on the WhatsApp communication platform. This media and communication platform was chosen since 64% of the Indonesian population currently use this platform and the users are relatively varied across age groups and other sociodemographic characteristics. The participants were recruited using a simplified-snowball sampling technique where invited candidate participants were requested to pass the invitations to their WhatsApp contacts. The minimum sample size was 1,068, based on the conservative assumption that the acceptability rate was 50 with a 3% margin of error and a confidence interval of 95%. To recruit the samples, participants were purposefully selected to include both urban and suburban areas.

The survey was estimated to take ~10 min to complete. To collect the information, a set of questions were constructed and developed. The questionnaire included sections on sociodemographic data, exposure to COVID-19 information, perceived risk of being infected with COVID-19, and acceptance of a vaccine. The questions were first pre-tested and were revised and finalized based on feedback from pre-testers.

### Study Variables

The response variable was acceptance of a hypothetical COVID-19 vaccine in Indonesian population. To assess the acceptance, the respondents were provided with the following information: (a) a vaccine is currently not available for COVID-19, but we want study participants to think about a hypothetical vaccine; (b) the hypothetical COVID-19 vaccine would be developed and tested clinically in humans; (c) clinical trials would show that the vaccine had a 5% chance of producing side effects like fever, skin rash and pain; and (d) the government would offer it as a free and optional vaccine. To assess the acceptance rate of the vaccine, the respondents were given two scenarios with different vaccine efficacies (95 and 50%). Participants were asked to respond to the question of whether they would be vaccinated with a new COVID-19 vaccine for each scenario (i.e., for 95 and 50%). The possible responses were “yes” or “no.”

Some explanatory variables were collected. Sociodemographic characteristics included age, gender, educational attainment, occupation, religion, marital status, monthly income, and type of urbanicity. Age was grouped into five categories (< 20, 21–30, 31–40, 41–50, and >51 years old); educational attainment was grouped into junior/senior school graduates, diploma graduates, and university graduates/post-graduates; and type of job was divided into five groups (civil servant, private sector employee, entrepreneur, student, and retired). Individual monthly income was grouped into < 2.5 million Indonesian Rupiah (IDR), 2.5–5 million, 6–10 million, and more than 10 million (< US$ 154.7, US$ 154.7–US$ 309.4, US$ 371.2–$ 618.8, and >US$ 618.8 using an April 4, 2020 exchange rate). Urbanicity of respondents was divided into rural and urban. Respondents were also asked whether they were working as a healthcare worker (HCW) or not and whether they had heard about COVID-19 prior to the survey. Their perceived risk of being infected with COVID-19 within the next month was assessed on a scale of 0 to 100% using a question based off previous studies (31, 32), where 0% indicates the lowest while 100% was the highest perceived risk. For statistical analysis the score was classified into five groups: 0, 10–20, 30–40, 50–60, and more than 60%.

### Statistical Analysis

A logistic regression model was employed to identify determinants of participants' acceptance of a COVID-19 vaccine. The analysis was conducted for both vaccine efficacies (i.e., 95 and 50%). In the first step, associations between explanatory variables and response acceptance were analyzed separately. In the second step, all variables with *p* ≤ 0.25 in the first step were included in the adjusted analysis. The significance of crude odds ratio (OR) from univariate analyses and adjusted OR (aOR) in multivariate analyses were assessed at α = 0.05. All analyses were performed using SPSS software (SPSS Inc., Chicago, IL, USA).

### Ethics Approval

The protocol of this study was approved by the Institutional Review Board of the School of Medicine, Universitas Syiah Kuala, Banda Aceh (041/EA/FK-RSUDZA/2020) and the National Health Research and Development Ethics Commission (KEPPKN) of the Ministry of Health of the Republic of Indonesia (#1171012P).

## Results

### Demographic Characteristics

We received 1,402 responses during the survey period; 43 of them were excluded due to incomplete data. More than half of the respondents (698/1,359; 51.4%) were among those aged 21–30 years old and 66.1% of them (898/1,359) graduated from a university (Table 1). Overall, 27.6% of respondents (375/1,359) worked in the private sector, 47.5% (645/1,359) earned < 2.5 million (equal to US$ 154.7) each month, and more than 75% (1041/1,359) lived in cities. Almost 40% (533/1,359) of the survey participants believed that they had a 0% risk of being infected with SARS-CoV-2.

**Table 1:** Unadjusted and adjusted logistic regression analyses showing factors associated with acceptance of a COVID-19 vaccine in Indonesia, 95% effectiveness (*n* = 1,359).

**Variable**	***n* (%)**	**Accept *n* (%)**	**Unadjusted**		**Adjusted**	
			**OR (95% CI)**	***p*–value**	**aOR (95% CI)**	***p*–value**
Age group (year)						
<20 *(R)*	201 (14.8)	192 (95.5)	1		1	
21–30	698 (51.4)	660 (94.6)	0.81 (0.39–1.71)	0.588	0.77 (0.32–1.87)	0.564
31–40	312 (23.0)	277 (88.8)	0.37 (0.17–0.79)	0.010	0.34 (0.11–1.02)	0.055
41–50	77 (5.7)	73 (94.8)	0.86 (0.26–2.86)	0.800	0.97 (0.22–4.22)	0.967
>51	71 (5.2)	66 (93.0)	0.62 (0.20–1.91)	0.404	1.22 (0.25–5.90)	0.801
Gender						
Male *(R)*	466 (34.3)	426 (91.4)	1		1	
Female	893 (65.7)	842 (94.3)	1.55 (1.01–2.38)	0.046	1.49 (0.95–2.35)	0.083
Educational attainment *(R)*						
Junior/senior school graduated	379 (27.9)	357 (94.2)	1		1	
Diploma graduated	82 (6.0)	73 (89.0)	0.50 (0.22–1.13)	0.096	0.48 (0.19–1.25)	0.133
University graduated/post-graduated	898 (66.1)	838 (93.3)	0.86 (0.52–1.43)	0.560	0.95 (0.49–1.82)	0.865
Occupation						
Civil servant *(R)*	270 (19.9)	248 (91.9)	1		1	
Private sector employee	375 (27.6)	351 (93.6)	1.30 (0.71–2.37)	0.396	1.16 (0.60–2.24)	0.651
Entrepreneur	186 (13.7)	171 (91.9)	1.01 (0.51–2.01)	0.974	1.18 (0.56–2.48)	0.668
Student	511 (37.6)	485 (94.9)	1.66 (0.92–2.98)	0.093	1.12 (0.47–2.69)	0.800
Retired	17 (1.3)	13 (76.5)	0.29 (0.09–0.96)	0.043	0.15 (0.04–0.63)	0.010
Religion						
Islam *(R)*	1229 (90.4)	1149 (93.5)	1		1	
Buddhism	41 (3.0)	39 (95.1)	1.36 (0.32–5.72)	0.677	1.01 (0.23–4.45)	0.988
Cristian	52 (3.8)	46 (88.5)	0.53 (0.22–1.29)	0.162	0.49 (0.19–1.24)	0.132
Catholic	28 (2.1)	25 (89.3)	0.58 (0.17–1.96)	0.381	0.48 (0.13–1.70)	0.253
Others	9 (0.7)	9 (100.0)	1 × 10^8^ (0.00–)	0.999	1 × 10^8^ (0.00–)	0.999
Marital status						
Single *(R)*	760 (55.9)	719 (94.6)	1		1	
Married	599 (44.1)	549 (91.7)	0.63 (0.41–0.96)	0.032	1.04 (0.53–2.04)	0.903
Monthly income (Indonesian Rupiah)						
<2.5 million *(R)*	645 (47.5)	604 (93.6)	1			
2.5–5 million	422 (31.1)	395 (93.6)	0.99 (0.60–1.64)	0.978		
6–10 million	195 (14.3)	179 (91.8)	0.76 (0.42–1.39)	0.370		
> 10 million	97 (7.1)	90 (92.8)	0.87 (0.38–2.01)	0.748		
Urbanicity						
Rural *(R)*	318 (23.4)	292 (91.8)	1		1	
Urban	1041 (76.6)	976 (93.8)	1.34 (0.83–2.15)	0.229	1.37 (0.83–2.27)	0.216
Healthcare related job						
No *(R)*	1095 (80.6)	1016 (92.8)	1		1	
Yes	264 (19.4)	252 (95.5)	1.63 (0.88–3.04)	0.123	2.01 (1.01–4.00)	0.048
Have heard about COVID-19						
No *(R)*	23 (1.7)	21 (91.3)	1			
Yes	1336 (98.3)	1247 (93.3)	1.33 (0.31–5.78)	0.700		
Perceived risk to be infected with COVID-19 (%)						
0 *(R)*	533 (39.2)	486 (91.2)	1		1	
10–20	374 (27.5)	353 (94.4)	1.63 (0.96–2.77)	0.074	1.39 (0.80–2.41)	0.241
30–40	180 (13.2)	170 (94.4)	1.64 (0.81–3.33)	0.167	1.52 (0.73–3.15)	0.262
50–60	227 (16.7)	217 (95.6)	2.10 (1.04–4.23)	0.038	2.21 (1.07–4.59)	0.032
>60	45 (3.3)	42 (93.3)	1.35 (0.40–4.54)	0.623	1.15 (0.33–3.97)	0.829

### Acceptance of COVID-19 Vaccine and Associated Variables

If the vaccine was 95% effective, 93.3% participants (1,268/1,359) would like to be vaccinated when it is provided freely by government. However, this percentage decreased to 67.0% (911/1,359) if vaccine efficacy was 50%.

In the first scenario, 95% effectiveness, an adjusted analysis found that being a HCW and having a higher perceived risk were associated with higher acceptance; being retired was associated with less acceptance compared to civil servants (Table 1). Those who were working as HCWs were twice as likely to accept a COVID-19 vaccine, aOR: 2.01; 95%CI: 1.01, 4.00, *p* = 0.048. In addition, those with a high score of perceived risk to be infected (50–60%) had twice the odds of vaccine acceptance compared to those with no perceived risk to be infected in the next month (aOR: 2.21; 95%CI:1.07, 4.59, *p* = 0.032). Those who were retired were less likely to accept the vaccine compared to those who were working as a civil servant, with the aOR: 0.15 (95%CI: 0.04, 0.63).

With a lower vaccine efficacy (50%), being a HCW was the only characteristic associated with vaccine acceptance. Those who were working as a HCW had 1.57 times greater odds of accepting the vaccine compared to those who were working in non-medical sectors, aOR: 1.57; 95%CI: 1.12, 2.20, *p* = 0.009 (Table 2).

**Table 2:** Unadjusted and adjusted logistic regression analyses showing factors associated with acceptance of a COVID-19 vaccine in Indonesia, 50% effectiveness (*n* = 1,359).

**Variable**	***n* (%)**	**Accept *n* (%)**	**Unadjusted**		**Adjusted**	
			**OR (95% CI)**	***p*–value**	**aOR (95% CI)**	***p*–value**
Age group (year)						
17–20 *(R)*	201 (14.8)	142 (70.6)	1		1	
21–30	698 (51.4)	477 (68.3)	0.90 (0.64–1.26)	0.534	0.88 (0.60–1.28)	0.499
31–40	312 (23.0)	196 (62.8)	0.70 (0.48–1.03)	0.069	0.69 (0.43–1.13)	0.144
41–50	77 (5.7)	47 (61.0)	0.65 (0.38–1.13)	0.126	0.71 (0.38–1.35)	0.302
> 51	71 (5.2)	49 (69.0)	0.93 (0.51–1.67)	0.796	1.14 (0.56–2.31)	0.722
Gender						
Male *(R)*	466 (34.3)	297 (63.7)	1		1	
Female	893 (65.7)	614 (68.8)	1.25 (0.99–1.59)	0.062	1.18 (0.93–1.51)	0.176
Educational attainment *(R)*						
Junior/senior school graduated	379 (27.9)	260 (68.6)	1			
Diploma graduated	82 (6.0)	55 (67.1)	0.93 (0.56–1.55)	0.787		
University graduated/post-graduated	898 (66.1)	596 (66.4)	0.90 (0.70–1.17)	0.438		
Occupation						
Civil servant *(R)*	270 (19.9)	176 (65.2)	1		1	
Private sector employee	375 (27.6)	250 (66.7)	1.07 (0.77–1.49)	0.695	1.05 (0.73–1.51)	0.787
Entrepreneur	186 (13.7)	122 (65.6)	1.02 (0.69–1.51)	0.929	1.12 (0.73–1.71)	0.599
Student	511 (37.6)	354 (69.3)	1.20 (0.88–1.65)	0.245	1.22 (0.79–1.90)	0.375
Retired	17 (1.3)	9 (52.9)	0.60 (0.22–1.61)	0.311	0.50 (0.18–1.43)	0.197
Religion						
Islam *(R)*	1229 (90.4)	826 (67.2)	1		1	
Buddhism	41 (3.0)	25 (61.0)	0.76 (0.40–1.44)	0.405	0.74 (0.39–1.43)	0.374
Cristian	52 (3.8)	36 (69.2)	1.10 (0.60–2.00)	0.761	1.16 (0.63–2.14)	0.625
Catholic	28 (2.1)	16 (57.1)	0.65 (0.31–1.39)	0.266	0.65 (0.30–1.40)	0.266
Others	9 (0.7)	8 (88.9)	3.90 (0.49–31.31)	0.200	4.32 (0.53–35.20)	0.172
Marital status						
Single *(R)*	760 (55.9)	510 (67.1)	1			
Married	599 (44.1)	401 (66.9)	0.99 (0.79–1.25)	0.950		
Monthly income (Indonesian Rupiah)						
<2.5 million *(R)*	645 (47.5)	439 (68.1)	1			
2.5–5 million	422 (31.1)	277 (65.6)	0.90 (0.69–1.16)	0.410		
6–10 million	195 (14.3)	131 (67.2)	0.96 (0.68–1.35)	0.817		
> 10 million	97 (7.1)	64 (66.0)	0.91 (0.58–1.43)	0.682		
Urbanicity						
Rural *(R)*	318 (23.4)	219 (68.9)	1			
Urban	1041 (76.6)	692 (66.5)	0.90 (0.68–1.17)	0.427		
Healthcare related job						
No *(R)*	1095 (80.6)	718 (65.6)	1		1	
Yes	264 (19.4)	193 (73.1)	1.43 (1.06–1.93)	0.020	1.57 (1.12–2.20)	0.009
Have heard about COVID-19						
No *(R)*	23 (1.7)	15 (65.2)	1			
Yes	1336 (98.3)	896 (67.1)	1.09 (0.46–2.58)	0.852		
Perceived risk to be infected with COVID-19 (%)						
0 *(R)*	533 (39.2)	345 (64.7)	1		1	
10–20	374 (27.5)	254 (67.9)	1.15 (0.87–1.52)	0.319	1.08 (0.81–1.44)	0.608
30–40	180 (13.2)	122 (67.8)	1.15 (0.80–1.64)	0.457	1.13 (0.78–1.63)	0.512
50–60	227 (16.7)	158 (69.6)	1.25 (0.89–1.74)	0.194	1.25 (0.89–1.76)	0.203
> 60	45 (3.3)	32 (71.1)	1.34 (0.69–2.62)	0.389	1.19 (0.61–2.36)	0.608

## Discussion

Vaccines are a key strategy to stop the escalation of the COVID-19 pandemic. As of April 8, 2020, there were more than 100 COVID-19 vaccine candidates being developed (33). This vaccine development is proceeding at a fast pace; prior to March 30, 2020, two vaccine candidates had entered Phase 1 clinical trials (13) while on April 9, five vaccine candidates in total were in Phase 1 clinical trials (33). In the region of southeast Asia, studies have been conducted to assess the acceptance of a vaccine against infectious diseases (21–26, 34). This present study was conducted to understand how the COVID-19 vaccine, when available, will be accepted by the general population in Indonesia, by asking individuals about a hypothetical vaccine—an approach used in many past studies (21, 26, 28–30). Understanding vaccine acceptance in Indonesia is important, given the large population and because the country has relatively high vaccine hesitancy for existing vaccines and relatively low vaccination coverage (18, 19). Characterizing how vaccine efficacy could impact acceptance is also important, given that actual or perceived vaccine efficacy could be relatively low.

Our findings indicated that when the vaccine is provided freely, 93.3 and 67.0% of participants would like to be vaccinated if the vaccine had 95% and 50% effectiveness, respectively. The acceptance rate for the first model (i.e., 95% efficacy) is far higher compared to acceptance of other new vaccines in southeast Asia (21, 22, 25, 34). This indicates that a majority of the general population in the country are supportive of the COVID-19 vaccine. This is not surprising because this study was started on March 25, 2020, when the number of COVID-19 cases started to sharply increase in Indonesia; 790 confirmed cases have been reported (35). It should be noted that the acceptance rate was measured under the presumption that the vaccine was provided freely by the government. Therefore, in the case that the vaccine needs to be purchased, or if it is not fully subsided by government, analyses assessing the acceptance at certain vaccine prices (i.e., willingness to pay) will need to be conducted not only in Indonesia but also in other countries in the southeast Asia region. We also note that it is unclear what the herd immunity threshold for COVID-19 is (36), and 67.0% vaccination coverage may be lower than what is required to stop the spread of disease.

Our study indicated that HCWs were more supportive of a COVID-19 vaccine than non-HCWs. Self-protection and desire to protect family, friends, and patients have been the drivers of HCWs' decision to get vaccinated in previous studies (37, 38). Since HCWs have more comprehensive knowledge about COVID-19, their relatively high awareness may lead them to protect themselves and not to transmit the virus to their family members. This might lead them to be more willing to accept the vaccine compared to those who working in non-medical sectors. In addition, our further analysis also suggested that the perceived risk of HCWs was higher compared to non-HCWs.

One important finding is that those who had a higher perceived risk to be infected with COVID-19 were more likely to accept the vaccine, but only for the 95% effective vaccine. Previous studies in Asia have found that perceived risk or perceived susceptibility to an infection is associated with positive support for vaccination (29, 30, 39). Another study also found that high perceived risk was associated with COVID-19 vaccine acceptance among general community members in Saudi Arabia (40) and among HCWs in China (41). Therefore, it is important to increase the perceived risk among communities since our study found that almost 40% of the respondents had a perceived risk of 0%. Low perceived risk may not only be correlated with vaccine acceptance, but also adherence to social distancing measures and other public health countermeasures. These relationships may be complicated—for example, an individual highly compliant with social distancing measures may perceive their risk to be low but still want to obtain a vaccine.

We also found that being retired had low acceptance compared to those who were working as civil servant. Lower vaccine acceptance among the retired population might be influenced by lower perceived risk. Although the elderly are more vulnerable to COVID-19, most of the retired population in Indonesia and indeed in southeast Asian countries have low mobility and spend more time at home with less travel. These behaviors may lead them to having a lower perceived risk of being infected with SARS-CoV-2, and eventually may lead to lower acceptance of a vaccine. Moreover, their acceptance might also be influenced by knowledge about the disease. Much of the information about COVID-19 is spread through social media or online media, which is less frequently accessed by older adults. Therefore, older adults might have less exposure to information about COVID-19 that could contribute to framing their risk perception. In addition, less social media use might also be associated with less knowledge among the elderly and this could affect their perceived risk and vaccine acceptance. However, this study did not measure respondents' knowledge of COVID-19 and we were unable to elucidate these relationships.

The study has several limitations. Generalizability of the survey results may be impacted by how we distributed the questionnaire. We used the WhatsApp platform, and so it may miss people from lower socioeconomic classes such as farmers, those with lower educational attainment, and those who were illiterate. According to UNESCO Indonesia, the literacy rate of adults (aged 15 and above) was 95.98% (42) and previous studies using community samples found that at least 96% of the community graduated from primary school (26, 43). As reported in other online studies in Indonesia (44–47), selection bias could also be related to the sampling technique and differential access to internet infrastructure across the country, as some regions have better internet access than others. Finally, acceptance was assessed using a hypothetical vaccine, which may differ from the respondents' revealed preferences in a real-life situation.

## Conclusion

Acceptance of the COVID-19 vaccine in Indonesia is influenced by the effectiveness of the vaccine. Acceptance is relatively high when the vaccine has a very high effectiveness, but it reduced to only 67.0% when the vaccine efficacy is 50%. If the COVID-19 vaccine has lower efficacy, governments will have to introduce more strategies to persuade their population to become vaccinated. In addition, since acceptance is associated with perceived risk for COVID-19, it is also important to increase the perceived risk in communities.

## Data Availability Statement

The datasets presented in this study can be found in online repositories. The names of the repository/repositories and accession number(s) can be found below: http://doi.org/10.6084/m9.figshare.12477143.

## Ethics Statement

The studies involving human participants were reviewed and approved by Institutional Review Board of the School of Medicine, Universitas Syiah Kuala, Banda Aceh (041/EA/FK-RSUDZA/2020) and National Health Research and Development Ethics Commission (KEPPKN) of the Ministry of Health of the Republic of Indonesia (#1171012P). The ethics committee waived the requirement of written informed consent for participation.

## Author Contributions

HH: conceptualization, methodology, software, data curation, formal analysis, resources, writing—original draft, writing—review and editing, project administration. AW: conceptualization, methodology, writing—review and editing. AY: methodology, writing—original draft, investigation, data curation, writing—review and editing, project administration. WW: methodology, investigation, project administration, data curation. SA: software, formal analysis, writing—review and editing. AG: investigation, project administration. AS: investigation. YR: writing—review and editing. HS: software, writing—review and editing. MM: project administration. All authors contributed to the article and approved the submitted version.

## Conflict of Interest

The authors declare that the research was conducted in the absence of any commercial or financial relationships that could be construed as a potential conflict of interest.
